# Both N- and C-terminal domains of galectin-9 are capable of inducing HIV reactivation despite mediating differential immunomodulatory functionalities

**DOI:** 10.3389/fimmu.2022.994830

**Published:** 2022-12-08

**Authors:** Ashwini Shete, Mahalakshmi Bhat, Jyoti Sawant, Supriya Deshpande

**Affiliations:** Division of Immunology and Serology, Indian Council of Medical Research (ICMR)-National AIDS Research Institute, Pune, India

**Keywords:** galectin-9, N- and C-terminal binding domains, HIV, reactivation, immunomodulation

## Abstract

**Background:**

The shock-and-kill strategy for HIV cure requires the reactivation of latent HIV followed by the killing of the reactivated cellular reservoir. Galectin-9, an immunomodulatory protein, is shown to induce HIV reactivation as well as contribute to non-AIDS- and AIDS-defining events. The protein is prone to cleavage by inflammatory proteases at its linker region separating the N- and C-terminal carbohydrate-binding domains (N- and C-CRDs) which differ in their binding specificities. It is important to study the activity of its cleaved as well as uncleaved forms in mediating HIV reactivation and immunomodulation in order to understand their role in HIV pathogenesis and their further utilization for the shock-and-kill strategy.

**Methodology:**

The PBMCs of HIV patients on virally suppressive ART (*n* = 11) were stimulated using 350 nM of the full-length protein and N- and C-CRDs of Gal-9. HIV reactivation was determined by analyzing gag RNA copies using qPCR using isolated CD4 cells and intracellular P24 staining of PBMCs by flow cytometry. Cytokine responses induced by the full-length protein and N- and C-CRDs of Gal-9 were also assessed by flow cytometry, Luminex, and gene expression assays. Changes in T helper cell gene expression pattern after the stimulation were also determined by real-time PCR array.

**Results:**

Both N- and C-CRDs of galectin-9 induced HIV reactivation in addition to the full-length galectin-9 protein. The two domains elicited higher cytokine responses than the full-length protein, possibly capable of mediating higher perturbations in the immune system if used for HIV reactivation. N-CRD was found to induce the development of Treg cells, whereas C-CRD inhibited the induction of Treg cells. Despite this, both domains elicited IL-10 secretory response although targeting different CD4 cell phenotypes.

**Conclusion:**

N- and C-CRDs were found to induce HIV reactivation similar to that of the full-length protein, indicating their possible usefulness in the shock-and-kill strategy. The study indicated an anti-inflammatory role of N-CRD versus the proinflammatory properties of C-CRD of galectin-9 in HIV infection.

## Introduction

Galectin-9 (Gal-9) is a soluble immunomodulatory β-galactoside-binding protein originally referred to as S-type lectin, widely expressed in different tissue cells and immune cells. The plasma levels of Gal-9 are elevated during infections like HIV, HCV, and tuberculosis (1, 2). The levels were shown to increase rapidly during acute HIV infection and remained elevated in chronic infection (3). Gal-9 levels were also shown to correlate positively with plasma HIV viral load in a number of studies (3–5). Endogenous levels of Gal-9 were shown to be associated with HIV transcription and HIV latency reversal (6). Further studies suggested that exogenous Gal-9 induces HIV transcription and reverses HIV latency *in vitro* and *ex vivo* (4). Gal-9 is known to cross-link several surface proteins and might modulate HIV transcription by different pathways. One of the studies reported the involvement of the TCR-dependent ERK and CREB signaling pathway (6), while another study suggested the modulation of gene expression levels of factors that regulate latency through interaction with N-linked oligosaccharides and O-linked hexasaccharides (4). Gal-9 interaction with its different receptors has been shown to have different outcomes in HIV infection. Gal-9:protein disulfide isomerase interaction on non-stimulated CD4^+^ T cells enhanced HIV entry and infection (7). In contrast, the interaction of Gal-9 with Tim-3 on PHA-stimulated CD4^+^ T cells caused a significant inhibition of HIV infection by both X4-tropic and R5-tropic isolates (8).

Its action as an immune modulator is also pleiotropic, contributing to tissue inflammation by activating macrophages and maturing dendritic cells on the one hand and inducing regulatory immune responses by promoting Treg cell and suppressing Th17 cell development on the other hand (9, 10). The varied activities of galectin-9 are the result of its structure constituted by N-terminal and C-terminal carbohydrate recognition domains (CRD) linked by a 14–56-amino acid polypeptide chain called a linker domain. It is a tandem repeat-type galectin as its N-terminal and C-terminal CRDs (N-CRD and C-CRD) are different, with only 39% amino acid homology leading to differences in their binding specificities (11). Regardless of the fact that the individual N- and C-terminals of Gal-9 CRDs have different oligosaccharide-binding affinities, they have in general shown to possess substantially lower eosinophil chemoattractant and hemagglutinin activities than full-length wild-type Gal-9 protein (12). The individual CRDs still have been shown to contribute differently to multiple functions in innate immunity and adaptive immunity (13) although some of the functions of the full-length Gal-9 are diminished. Studies have shown that Gal-9 C-CRD exhibits a greater antiproliferative and pro-apoptotic activity than Gal-9 N-CRD (14). Gal-9 N-terminal is efficient in activating dendritic cells (DCs), whereas C-terminal oligomerization contributes to T-cell death (13).

The linker region of galectin-9 is susceptible to proteolytic cleavage by extracellular proteases like MMP-9, elastase, etc. leading to the dissociation of individual CRDs altering the functionality of the full-length protein (15). Since HIV infection is characterized by inflammatory responses and HIV proteins have been shown to induce MMP-9 activation (16, 17), it is likely that HIV-infected individuals harbor cleaved Gal-9 protein with dissociated N- and C-terminal CRDs. Although the full-length galectin-9 has been used for HIV reactivation experiments until now, the effect of individual CRDs on HIV reactivation is not yet determined. It is important to determine if the individual CRDs can also contribute to HIV reactivation and immunomodulation in addition to their full-length counterpart to understand their role in HIV pathogenesis. Hence, we conducted a study to determine the HIV reactivation potential of individual CRDs of Gal-9, and we also determined the effect of these CRDs on T-cell responses.

## Methodology

### Study population

The study was conducted at ICMR-National AIDS Research Institute (NARI), India, after the approval of the study protocol by the Institutional Ethics Committee (Protocol No. NARI-EC/2017-13). The study was conducted on stored peripheral blood mononuclear cells (PBMCs) isolated from virally suppressed HIV-infected patients from antiretroviral therapy (ART) centers located in Pune City at 1 year after the initiation of antiretroviral therapy (*n* = 12; M:F = 4:8). All the patients were on first-line ART regimen consisting of two nucleotide reverse transcriptase inhibitors and one non-nucleoside reverse transcriptase inhibitor as per the national program of the country prevailing at the time of the enrollment. Blood samples of the patients were collected after obtaining their written informed consent. The median age of the participants was 40 (range: 27 to 60) years, and the median CD4 count was 346.5 (range: 244 to 861) cells/mm^3^. All the patients were treatment responders as indicated by their undetectable viral loads. The PBMCs of HIV-uninfected individuals (*n* = 3) were used as controls for intracellular P24 expression.

### Gal-9 proteins used in the experiments

Full-length and cleaved galectin-9 proteins were obtained from a commercial source. Full-length Gal-9 protein (R&D Laboratories, USA, accession number: NP002299) was a 36-kDa protein expressed in human embryonic kidney cells (HEK293) having >95% purity as assessed by SDS-PAGE visualized with silver staining and quantitative densitometry by Coomassie blue staining. The endotoxin level was below 0.10 EU per 1 μg of the protein by the LAL method. The N-terminal (purchased from Novus Biologicals, USA) and C-terminal CRDs (synthesized by Bhat Biotech, India) spanned 1–148 (18.5 kDa) and 174–323 (16.4 kDa) amino acid regions on the N- and C-terminus of the full-length Gal-9 sequence, respectively. They were expressed in *Escherichia coli* vector and had a purity of >90% as assessed by SDS-PAGE.

### Incubation with exogenous Gal-9 and determination of cytotoxicity

PBMCs (1 *×* 10^6^) were treated with three concentrations (200, 350, and 500nM) of the full-length Gal-9 protein (R&D Laboratories, USA) for 24 h at 37°C. The cytotoxicity of the Gal-9 was determined by flow cytometry using LIVE/DEAD (Fixable violet Dead cell Stain Kit, for 405 nm excitation, Thermo Scientific, USA) and Annexin V-PE (BD Biosciences, USA) which detects apoptotic cells. Annexin V expression was also assessed at 48 h after treatment with 350 nM of Gal-9 full-length, N-terminal, and C-terminal CRD proteins.

### Effect of exogenous Gal-9 on HIV reactivation in the J-Lat 6.3 cell line

The effect of varying concentrations starting from a low dose of 50 to 350 nM of the full-length, N-terminal, and C-terminal CRD proteins on HIV reactivation in the J-Lat model of HIV latency was determined using the J-Lat 6.3 cell line. The cells were incubated with varying concentrations of the constructs for 24 h. HIV reactivation was assessed by GFP expression using FACS Fusion I (BD Biosciences, USA). The data analysis was done by using FACSDiva software version 9.0.1 and FlowJo version 8.0.3.

### Flow cytometry analysis of the reactivated HIV-infected PBMCs

Exogenous full-length, N-terminal, and C-terminal CRDs at a concentration of 350 nM were incubated with 1 × 10^6^ PBMCs of HIV-infected patients in a humidified 5% CO_2_ incubator at 37°C for 24 h. Cells were stained with antibodies against surface markers like CD3 APC H7, CD8 BUV737, Tim-3 PECF594, CD45RA PECy5, and CCR7 BUV395 (BioLegend and BD Biosciences, USA) antibodies for 30 min at room temperature. The cells were permeabilized (Permeabilizing Solution II, BD Biosciences, USA) and then stained with an intracellular antibody cocktail for 30 min at room temperature against cytokines and proliferation markers interleukin (IL)-2 BV605, IL-17a BV510, IL-10 PE, interferon (IFN)-γ PECy7, Ki-67 BV786, and FITC P24 (KC-57) (BioLegend, BD Biosciences, Beckman Coulter, USA). The cells were acquired on FACS Fusion I (BD Biosciences, USA) within 24 h to get 50,000 of the gated events of lymphocytes. The data analysis was done by using FACSDiva software version 9.0.1 and FlowJo version 8.0.3.

### CD4^+^ T-cell enrichment from the patients’ PBMCs

CD4^+^ T cells were enriched from the PBMCs of HIV-infected patients (*n* = 3) using MojoSort™ Human CD4 T-Cell Negative Isolation Kit (BioLegend, USA). The enriched CD4 T cells (1 × 10^6^ per condition) were incubated overnight with exogenous Gal-9 full-length (350 nM), N-terminal (350 nM), and C-terminal CRDs (350 nM) at 37°C for analyzing the expression of HIV gag copies and host gene expression by T helper activation PCR array (RT profiler PCR arrays).

### HIV gag RNA quantification by qPCR

After stimulation with the full-length, N-terminal, and C-terminal CRDs of Gal-9 for 6 h, RNA extraction was performed (Applied Biosystems, USA) from CD4 cells. The double-stranded cDNA construct was prepared using a cDNA synthesis kit (Thermo Fisher Scientific, USA). cDNA was further used for real-time PCR on 7500HT FAST Real-Time PCR System with 96-well module (Applied Biosystems, Thermo Fisher Scientific, USA) using SYBR Green (Applied Biosystems, Thermo Fisher Scientific, USA) for determining gag copies as described previously (18). The sequences of gag forward and reverse primers were (5′ to 3′) ACCCATGTTTACAGCATTATCAGAAG and GCTTGATGTCCCCCTACTGTATTT, respectively. The *β-actin* gene was used as the internal/housekeeping gene to semiquantitate the gag gene expression. Fold change 2^−ΔΔCt^ was calculated by dividing CT values in Gal-9-treated cells by unstimulated controls after normalizing them by subtracting CT values of the housekeeping gene.

### Determination of cytokine secretion pattern after Gal-9 treatment

The supernatants of PBMCs treated with the full-length, N-terminal, and C-terminal CRDs of Gal-9 were processed for the detection of cytokine profile using the Luminex assay (Bio-Rad Laboratories, USA) according to the manufacturer’s instructions. The cytokines assessed were IFN-γ, IL-10, IL-17, and tumor necrosis factor (TNF)-α. The plates were read using the Bio-Plex 200 system and analyzed using the Bio-Plex Manager software (Bio-Rad Laboratories, USA).

### T helper activation profile after Gal-9 treatment

RNA was extracted from the stimulated CD4^+^ T cells (Applied Biosystems, USA), and cDNA was prepared using an RT2 first-strand kit (Qiagen, Germany). T helper activation RT profiler PCR array was used for determining the T helper cell activation profile using an RT^2^ Real-Timer SyBR Green qPCR master mix (Qiagen, Germany) and ABI 7500HT system (Applied Biosystems, USA). Data were analyzed using a data analysis tool provided by the manufacturer. Fold change 2^−ΔΔCt^ was calculated by dividing CT values in Gal-9-treated cells by unstimulated controls after normalizing them by subtracting the average CT values of the housekeeping genes used in the array. *p*-values were calculated by software using Student’s *t*-test for all the genes.

### Data analysis

The percentages of different markers expressed by T cells after stimulation with the full-length, N-terminal, and C-terminal CRDs of Gal-9 were compared with the unstimulated cells. The Wilcoxon signed-rank non-parametric hypothesis test was used to determine the significant differences. Similarly, the levels of the cytokines secreted in supernatants of the stimulated cells assessed by the Luminex assay were compared by the Wilcoxon signed-rank non-parametric hypothesis test. All *p*-values are two-tailed unless specified otherwise.

## Results

### Determination of non-cytotoxic concentration of Gal-9

**Figure 1:**
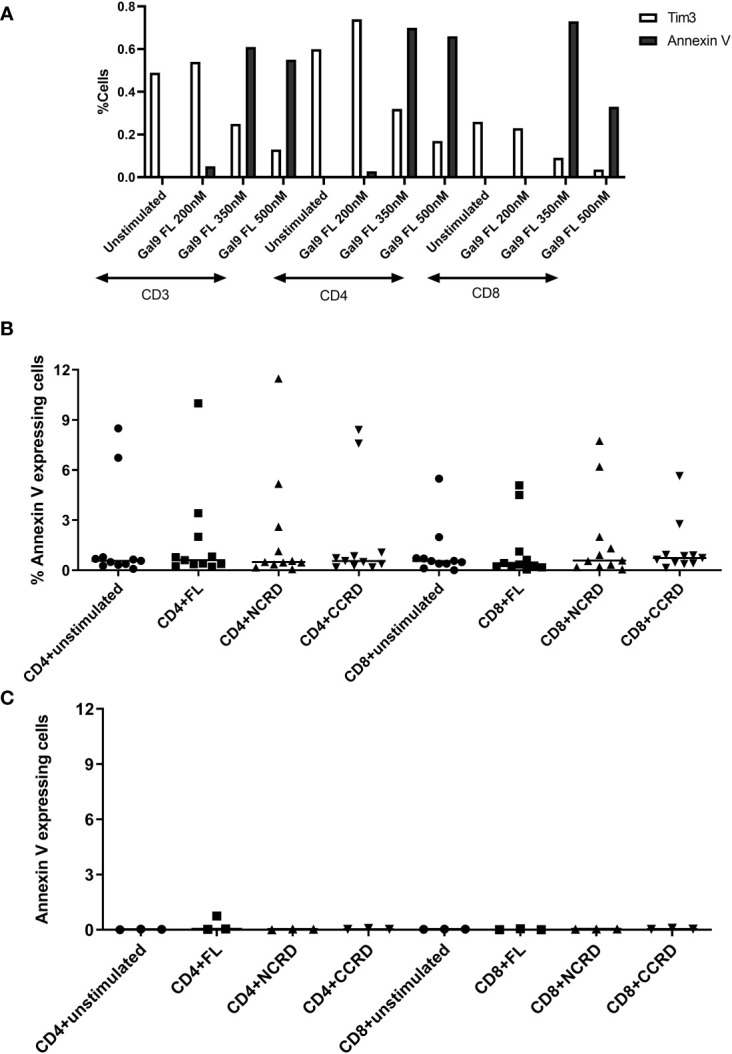
Expression of Annexin V and Tim-3 at different concentrations of the full-length and individual CRDs measured by flow cytometry assay. **(A)** CD3, CD4, and CD8 cells expressing Annexin V and Tim-3 after stimulation of peripheral blood mononuclear cells (PBMCs) with 200, 350, and 500 nM of full length Gal-9. **(B, C)** Percentage of CD4 and CD8 cells expressing Annexin V when PBMCs were stimulated with 350 nM concentration of all three fractions for 24 [**(B)**, *n* = 11] and 48 [**(C)**, *n* = 3] h. Individual values are plotted as scatter plots, and the horizontal line represents median values.

PBMCs stimulated with the full-length, N-terminal, and C-terminal CRDs of Gal-9 at three different concentrations (200, 350, and 500 nM) showed more than 98% viability which was similar to the unstimulated condition. Furthermore, simultaneous Annexin V and Tim-3 expression was also assessed after treatment of PBMCs with the three concentrations of the full-length Gal-9 to determine the non-apoptotic concentration of Gal-9. Annexin V expression on CD4 and CD8 T cells increased with the increasing concentrations of the full-length Gal-9 from 200 to 350 nM, whereas Tim-3 expression was found to be diminished (**Figure 1A**), suggesting the involvement of the Tim-3 receptor in inducing T-cell apoptosis by the full-length Gal-9. The concentration of 350 nM for the full-length as well as N- and C-CRDs was chosen for all further experiments to determine the effect of these forms on HIV reactivation and T-cell responses as it showed a moderate effect on inducing apoptosis. Annexin V expression was also assessed after the treatment of PBMCs with 350 nM of all three forms. Median Annexin V expression was found to be less than 12% as shown in **Figures 1B, C** after treatment with all three forms at 24 and 48 h. Annexin V expression did not differ significantly after stimulation with any of the forms.

### HIV reactivation after stimulation with the full-length and individual Gal-9 CRDs

**Figure 2:**
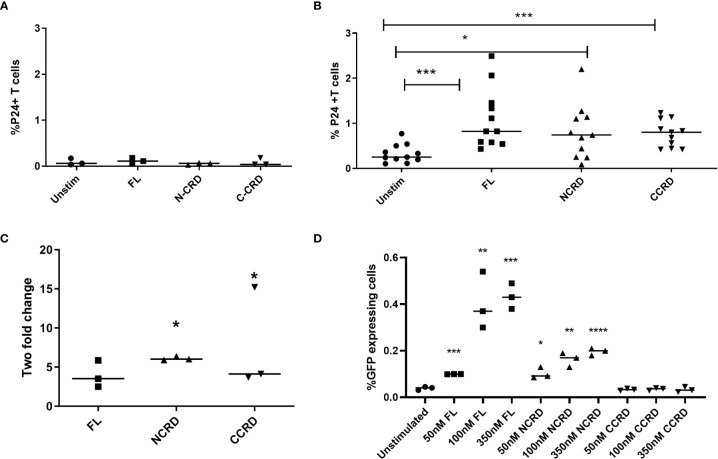
HIV reactivation after stimulation with the full-length and individual CRDs measured by flow cytometry for detecting intracellular P24 expression **(A)** in PBMCs of HIV-negative individuals used as controls (*n* = 3) and **(B)** in PBMCs of HIV-infected individuals on virally suppressive first-line ART (*n* = 11). **(C)** HIV reactivation was assessed by RT-qPCR showing fold change in HIV gag gene expression after stimulation of enriched CD4 cells with the full-length Gal-9 and individual CRDs (*n* = 3). Individual values are plotted as scatter plots, and the horizontal line represents median values. *p*-values showing significant differences (*p* < 0.05) between unstimulated and treated cells as calculated by the Wilcoxon signed-rank test are shown. **(D)** HIV reactivation assessed using the J-Lat *in-vitro* model of HIV latency after stimulation of J-Lat 6.3 cells with 50, 100, and 350 nM of the full-length, N-CRD, and C-CRD Gal-9 constructs. Individual values are plotted as scatter plots with the horizontal line representing median values. Statistical comparisons were performed using two-tailed unpaired *t*-tests. *p < 0.05, **p < 0.01, ***p < 0.001, ****p < 0.0001.

HIV reactivation after stimulation with the full-length and individual Gal-9 CRDs was assessed by intracellular P24 expression by flow cytometry. Stimulation with the full-length, N-terminal, and C-terminal CRDs of Gal-9 escalated the P24 expression significantly (*p* = 0.0010, 0.0137, and 0.0010, respectively) in CD4 T cells as compared with the baseline expression in unstimulated cells of HIV-infected patients, but not of HIV-uninfected individuals as shown in **Figures 2A, B**. Full-length and C- and N-terminal CRDs of galectin-9 were also tested for HIV reactivation in enriched CD4 T cells from HIV-infected patients (*n* = 3) by real-time PCR assay. HIV reactivation was observed with all three stimulants as indicated by the fold change for HIV gag copies as shown in **Figure 2C**. HIV reactivation observed in the J-Lat 6.3 cell line is shown in **Figure 2D**. Full-length and N-CRD constructs induced HIV reactivation in the J-Lat model of HIV latency at concentrations of 50, 100, and 350 nM (*p* = 0.0001, 0.007, and 0.0003 and *p* = 0.0115, 0.0024, and <0.0001, respectively). However, C-CRD did not induce any reactivation in the J-Lat 6.3 cells.

### CD4 and CD8^+^ T-cell cytokine responses induced by Gal-9 as assessed by flow cytometry

**Figure 3:**
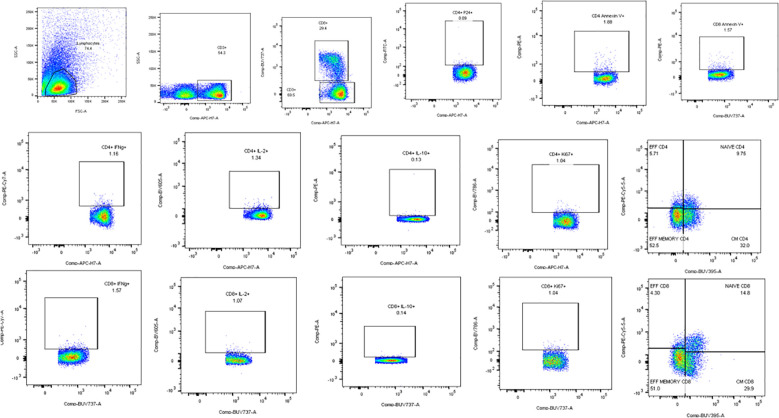
Representative flow cytometry plots showing the gating strategy used for the analysis of the flow assays. Lymphocytes identified by forward and side scatter were further gated for the identification of CD3, CD4, and CD8 T cells. All the other markers were plotted on CD4 and CD8 cells as shown in the plots.

**Figure 4:**
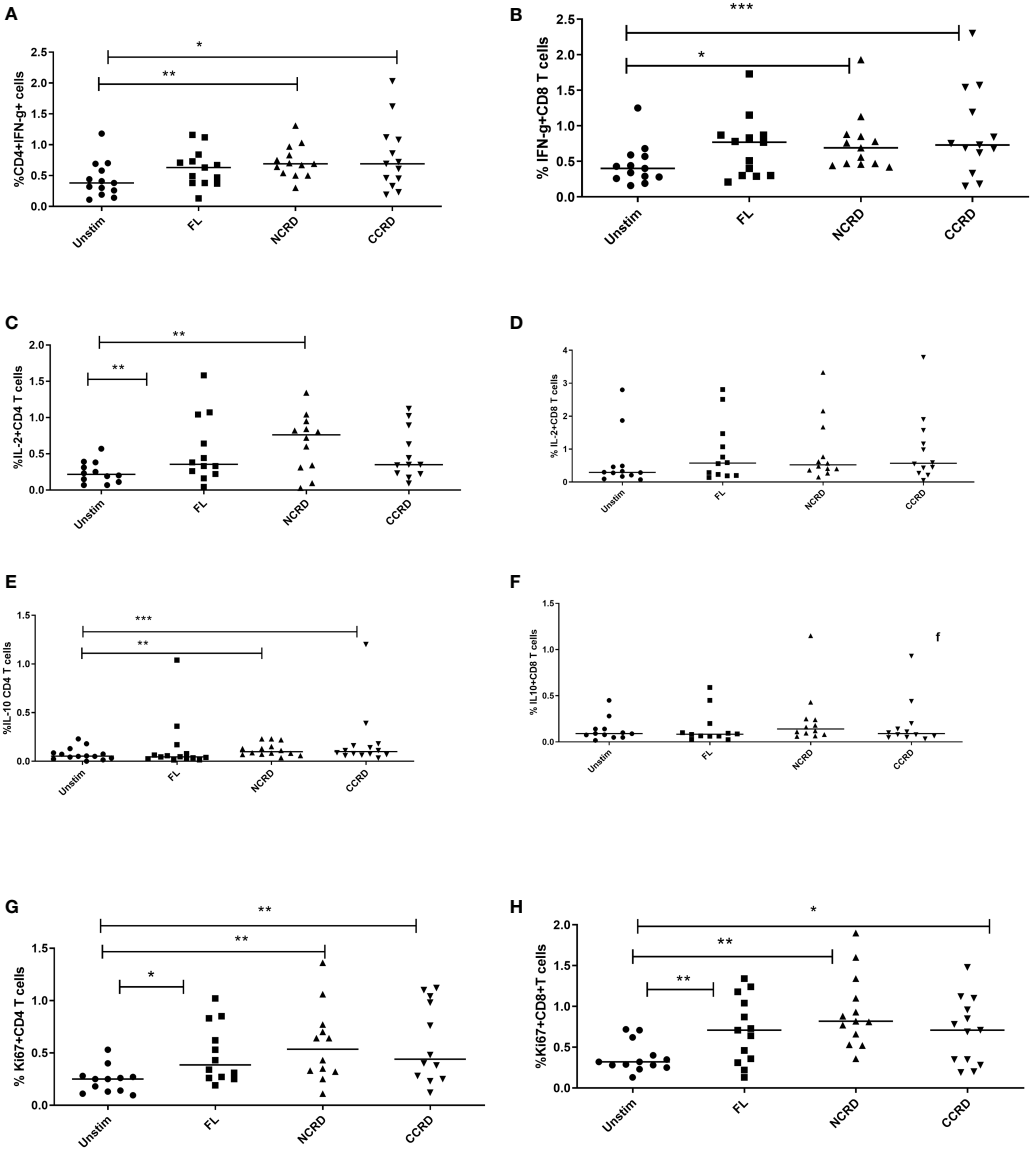
Cytokine response after treatment with the full-length and individual Gal-9 CRDs (*n* = 11). The cells were acquired on BD FACS Aria Fusion, and data were analyzed by using FlowJo V10 after staining with fluorochrome-labeled antibodies against IFN-γ, IL-2, IL-10, and Ki-67. The % expression of **(A)** IFN-γ in CD4 cells, **(B)** CD8 cells expressing IFN-γ, **(C)** IL-2 in CD4 cells, **(D)** IL-2 in CD8 cells, **(E)** IL-10 in CD4 cells, **(F)** IL-10 in CD8 cells, **(G)** Ki-67 in CD4 cells, and **(H)** Ki-67 in CD8 cells is shown. Individual values are plotted as scatter plots, and the horizontal line represents median values. *p*-values showing significant differences (*p* < 0.05) between unstimulated and treated cells as calculated by the Wilcoxon signed-rank test are shown. *p < 0.05, **p < 0.01, ***p < 0.001.

Flow cytometry assays were done to determine the effect of the full-length and individual Gal-9 CRDs on the expression of different cytokines and surface markers on CD4 and CD8 T cells. The gating strategy used for the flow cytometry assays is shown in **Figure 3**. IFN-γ cytokine expression in CD4 and CD8 T cells (**Figures 4A, B** **)** significantly increased when stimulated with the C-terminal (*p* = 0.0176 and 0.0010, respectively) and N-terminal CRDs (*p* = 0.006 and 0.0117, respectively). No significant difference was detected in IFN-γ-expressing T cells stimulated with the full-length Gal-9 and unstimulated cells. A notable rise in IL-2 expression by CD4 T cells (**Figures 4C, D** **)** was observed when stimulated with the full-length Gal-9 (*p* = 0.0068) and N-terminal CRD (*p* = 0.0029). IL-10 cytokine expressing CD4 T cells (**Figures 4E, F** **)** increased remarkably when stimulated with the N- and C-terminal CRDs of Gal-9 (*p* = 0.009 and 0.0001, respectively), while IL-2 and IL-10 expressing CD8 T cells showed no significant differences. The frequency of CD4 and CD8 T cells expressing the proliferation marker, Ki-67, was significantly high after stimulation with the full-length (*p* = 0.0208 and 0.0093, respectively), N-terminal (*p* = 0.0053 and 0.0015, respectively), and C-terminal (*p* = 0.0086 and 0.0408, respectively) CRDs as compared with the unstimulated cells (**Figures 4G, H** **)**.

### Analysis of the CD4 phenotype responsible for IL-10 expression

**Figure 5:**
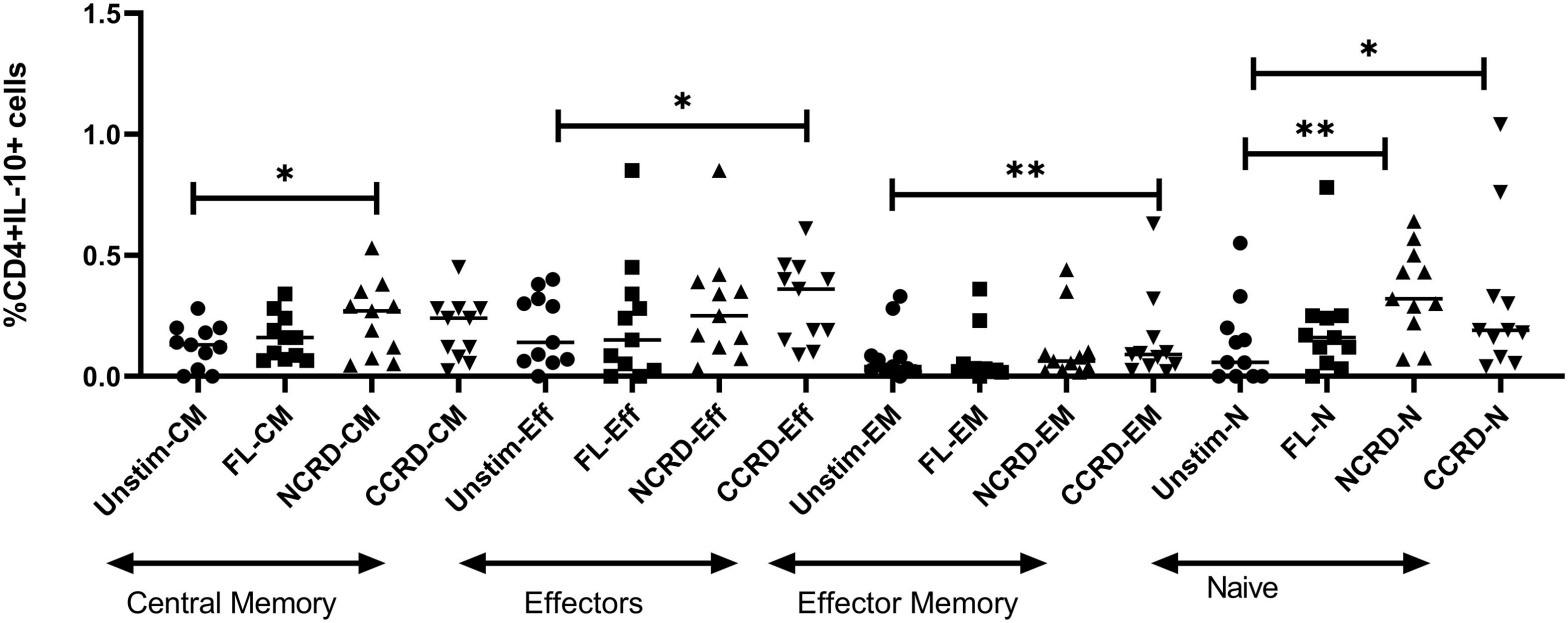
IL-10 expression by central memory, effector, effector memory, and naive CD4 identified based on CCR7 and CD45RA expression using intersecting gates after treatment with the full-length, N-CRD, and C-CRD Gal-9 proteins (*n* = 11). The percentage of CD4 cells expressing IL-10 is shown on the *Y*-axis, and different CD4 phenotypes and conditions used are shown on the *X*-axis. Individual values are plotted as scatter plots, and the horizontal line represents median values. *p*-values showing significant differences (*p* < 0.05) between unstimulated and treated cells as calculated by the Wilcoxon signed-rank test are shown. *p < 0.05, **p < 0.01.

We analyzed the CD4 phenotype responsible for IL-10 secretion by flow cytometry using CD45RA and CCR7 expression. CD45RA^+^CCR7^+^ CD4 cells were classified as naive, CD45RA^+^CCR7^−^ as effectors, CD45RA^−^CCR7^+^ cells as central memory, and both negative cells were considered as effector memory CD4 cells. The N-CRD fraction caused a significant induction of IL-10 response from central memory and naive cells, whereas the C-CRD part induced a significant IL-10 expression by effectors, effector memory, and naive cell population (**Figure 5**), indicating differences in the target cell populations for these two fractions.

### Cytokine responses induced by Gal-9 as assessed by multiple Luminex assay

**Figure 6:**
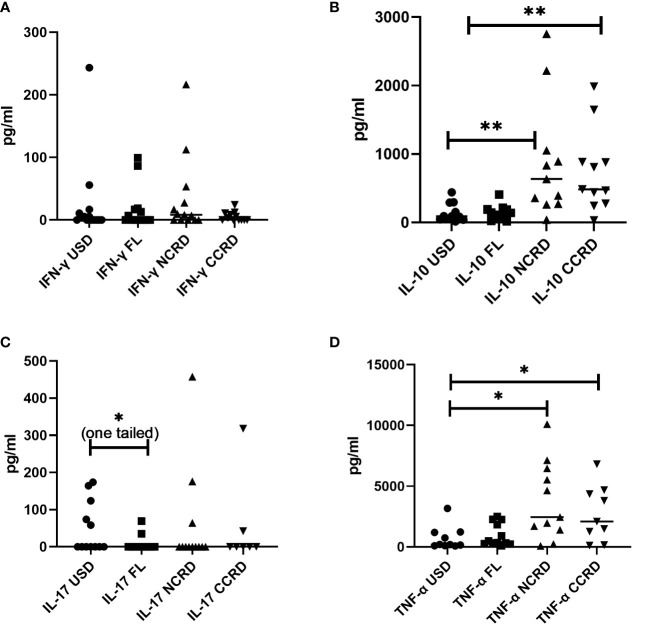
The levels of **(A)** IFN-γ, **(B)** IL-10, **(C)** IL-17, and **(D)** TNF-α (pg/ml) plotted on the *Y*-axis in the supernatants of PBMCs stimulated with the full-length Gal-9 and individual CDRs (shown on the *X*-axis) as assessed by the Luminex assay. Individual values are plotted as scatter plots, and the horizontal line represents median values. *p*-values showing significant differences (*p* < 0.05) between unstimulated (USD) and treated cells as calculated by the Wilcoxon signed-rank test are shown. *p < 0.05, **p < 0.01.

Contrary to the significant responses observed by flow cytometry, IFN-γ secreted by PBMCs did not show significant differences as compared with the unstimulated controls (**Figure 6**). However, N-CRD (*p* = 0.0043) and C-CRD (*p* = 0.0017) induced significant IL-10 secretory responses as observed by flow cytometry. TNF-α secretion was also higher after N-CRD (*p* = 0.039) and C-CRD (*p* = 0.046) treatment. IL-17 secretion decreased significantly (*p* = 0.03, one-tailed) in the full-length-treated PBMCs as compared with their unstimulated controls.

### Differential T helper cell activation profile induced by the full-length, N-terminal, and C-terminal CRDs of galectin-9

**Figure 7:**
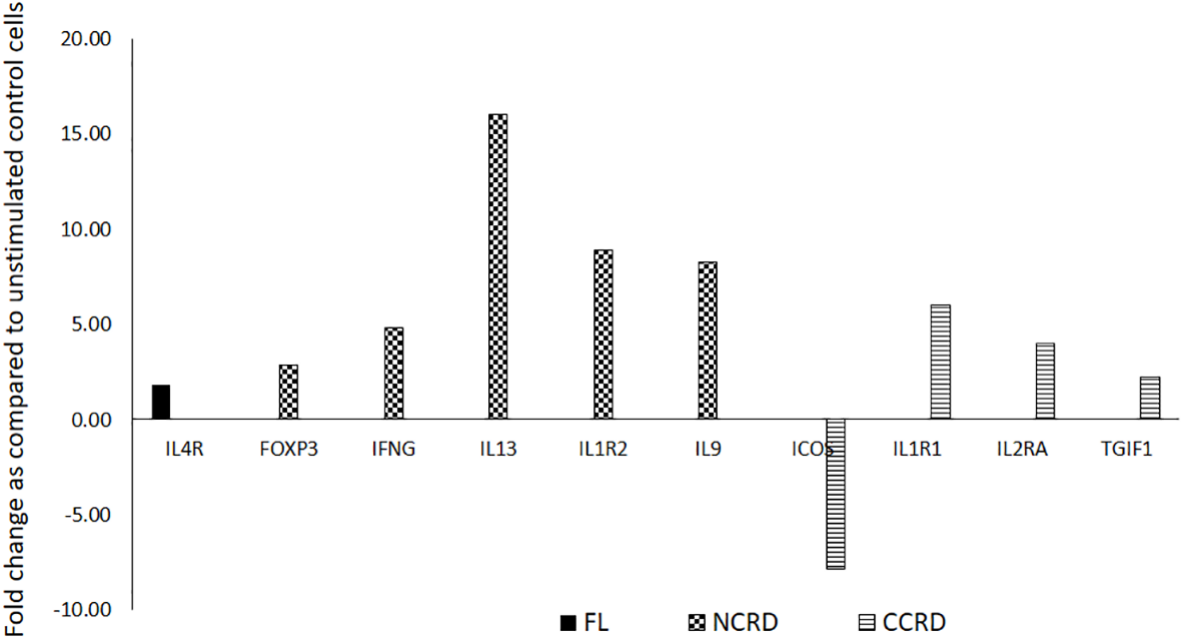
T helper cell activation profile induced by the full-length and individual CRDs of galectin-9 using T helper activation RT profiler PCR array and ABI 7500HT system. Genes showing significant up- or downregulation after treatment with the full-length, N-CRD, and C-CRD Gal-9 proteins are shown.

We further assessed the gene expression profile associated with T helper cell activation after stimulating enriched CD4 T cells with the full-length as well as C- and N-terminal CRDs of galectin-9 in three independent experiments. The genes showing significant alteration in expression levels as compared with the unstimulated control are shown in **Figure 7**. Full-length Gal-9 stimulation resulted in significant upregulation of IL-4R (*p* = 0.021), whereas N-terminal CRD stimulation resulted in significant upregulation of FoxP3 (*p* = 0.039), IFN-γ (*p* = 0.035), IL-13 (*p* = 0.016), IL-1R2 (*p* = 0.049), and IL-9 (*p* = 0.0014) gene expression. C-terminal CRD stimulation resulted in significant upregulation of IL-1R1 (*p* = 0.0087), IL-2RA (*p* = 0.0005), and TGIF1 (*p* = 0.027) and downregulation of ICOS (*p* = 0.007).

## Discussion

Galectin-9 is known to play a complex immunomodulatory role either by inhibiting or promoting various phases of the host immune responses and to mediate multiple complex signaling events that affect T cells in both immunosuppressive and inflammatory manner. These differing functionalities are mediated by its two heterologous CRDs through their interaction with several ligands like Tim-3, CD44, and protein disulfide isomerase (7, 19, 20). There is a report suggesting that the two Gal-9 CRDs might interact differently with the same receptor (13) adding to its complex functionality. Gal-9 has the potential to influence the course of HIV infection as it can promote tissue inflammation and T-cell exhaustion as well as alter HIV infection through engagement with the Tim-3 receptor and PDI. Moreover, cleaved Gal-9 levels were also shown to be elevated in HIV infection as well as to be correlated with inflammatory markers and disease progression (21). Hence, we planned to study the role of individual CRDs in HIV reactivation to determine their potential to act as latency-reversing agents. While evaluating any LRA, it is required to assess if it also mediates other functions which potentially interfere with the immune elimination of the reactivated reservoir or could lead to hyperimmune activation and contribute to its adverse reactions. Hence, we also determined T-cell functionalities mediated by these CRDs.

Gal-9 has been shown to trigger activation or apoptosis of CD4 helper cells at 5–30 nM concentrations (22). However, the studies reporting HIV reactivation have used concentrations ranging from 200 to 1,000 nM (4, 6), possibly indicating that higher concentrations of Gal-9 might be required for the reactivation of HIV. Hence, we also evaluated 200–500 nM concentrations to stimulate primary cells in our assays as our primary aim was to determine its effect on HIV reactivation and to check its effects on T-cell functions while it reactivates HIV. Although these concentrations are much higher than the plasma levels, the concentrations at the tissue levels or in the vicinity of cells secreting it where it is likely to mediate most of its action might differ from the plasma levels. We also evaluated the effect of lower doses starting from 50 nM of the constructs in inducing HIV reactivation in the J-Lat model of HIV latency with GFP used as a reporter. While we observed a significant increase in GFP expression at concentrations ranging from 50 to 350 nM of the full-length and N-CRD Gal-9 constructs, we failed to detect reactivation by any of the used concentrations of the C-CRD construct in the J-Lat 6.3 cell line. The level of GFP expression was observed to be low after stimulation with the full-length and N-CRD although it was significantly higher than the unstimulated control. Different clones of J-Lat have been shown to respond differently to exogenous Gal-9, and low-level reactivation has been reported in J-Lat 6.3 in one of the previous studies (4, 6). We did observe apoptosis in not more than 12% of our cell population as also reported by one of the studies previously (6). Gal-9 induces apoptosis or activation in distinct subsets of human T cells using two distinct signaling pathways (23) which in turn would depend on the expression pattern of its receptors. Simultaneous PD-1 expression on T cells has been shown to attenuate cell death induced by Gal-9/Tim-3 interaction (24), indicating the complex interplay of Gal-9 with its ligands.

N- and C-terminal CRDs were found to induce HIV reactivation along with the full-length Gal-9 in the PBMCs of HIV-infected individuals on virally suppressive ART, indicating that cleavage of the full-length Gal-9 may not be associated with the loss of its effect on HIV reactivation. Their HIV reactivating ability might also contribute to enhanced disease severity observed in the presence of higher cleaved Gal-9 levels (21). We also observed whole Gal-9 levels consisting of cleaved and full-length Gal-9 protein to be associated more strongly with HIV viral load than the full-length Gal-9 (data not published). Earlier studies for HIV reactivation were done using the stabilized form of the full-length Gal-9 where cleavage at the linker region was prevented (4, 6). We report here, for the first time, HIV reactivation by the individual CRDs. HIV reactivation was assessed in CD4^+^ T cells which are known to harbor the predominant replication-competent HIV reservoir (25). However, we did not assess if the induced HIV was replication-competent. Instead, we limited our assays to assess if the virus is transcription- and translation-competent as such reservoir also contributes to HIV pathogenesis and needs to be targeted (26). There was no significant difference observed in the potency of these three forms in reactivating HIV. In contrast, the two CRDs induced the secretion of more cytokines than the full-length Gal-9 protein. The protease cleavage of FL-Gal-9 has been shown to lead to uncontrolled hyperimmune activation, including a cytokine storm (15). N- and C-CRDs were observed to induce IFN-γ, IL-10, and TNF-α cytokine responses by flow cytometry and/or Luminex assay. The interaction of Tim-3 with galectin-9 had been shown to enhance IFN-γ production by NK cells in one of the studies (27), although it has been shown to impair NK cell functionality in other studies (28, 29), again pointing out the complexity of interactions of Gal-9 with its ligands in mediating activating or inhibitory functions likely to be influenced by the surrounding microenvironment (27). Gal-9 has also been shown to promote TNF production from the microglia although in a Tim-3-independent manner (30).

The interaction of Gal-9 with its receptor, CD44, has been shown to upregulate FoxP3 expression in induced regulatory T cells (iTreg), which is further responsible for the production of IL-10 by these cells (19). We also observed higher IL-10 levels in N- and C-CRD-stimulated PBMCs as well as upregulation of FoxP3 gene expression in CD4^+^ T cells after N-CRD stimulation. Interestingly, downregulation of ICOS and upregulation of TGIF1 through C-terminal CRD stimulation were observed suggesting the possible suppression of Tregs after C-CRD treatment. Despite this, it increased IL-10 secretion from PBMCs and T cells. FoxP3-negative Th1 cells (31) as well as CD8 (32) have also been shown to serve as a source of IL-10. When we investigated into the type of cells responsible for IL-10 secretion after stimulation with N- and C-CRDs, we observed that N-CRD was responsible for IL-10 secretion by central memory cells as against C-CRD, which induced IL-10 secretion by effectors and effector memory cells. HIV reactivation has also been shown to be accompanied by CD44 upregulation (33). We had previously shown that HIV-infected cells express IL-10 after HIV reactivation (34). The link between Gal-9, HIV reactivation, and IL-10 secretion through CD44 signaling needs to be investigated further.

C-terminal CRD stimulation in our study showed the upregulation of the expression of IL-1R1 (immunoinflammatory), an important mediator involved in many cytokine-induced immune and inflammatory responses, whereas the IL-1R2 gene expression, which acts as a decoy receptor (35) inhibiting the activity of its ligands, was upregulated by N-terminal CRD stimulation. Stimulation with N-terminal CRD also upregulated the anti-inflammatory cytokines like IL-13 and IL-9 shown to be secreted by Treg (36, 37). As against the anti-inflammatory portfolio of N-CRD, C-CRD seemed to possess proinflammatory properties by upregulating IL-2RA, the most prominent T-cell activation marker, although it is shown to be expressed by Treg cells (38). Blockade of ICOS signaling has been shown to increase CD25 expression without affecting IL-10 production in one of the studies (39). Despite the differential effect of N- and C-CRDs on the induction of Treg cells observed in the study, the cytokine secretion pattern induced by them did not seem to vary much. TNF-α, a proinflammatory cytokine, was also found to be secreted after N-CRD treatment. TNF-α secreting Treg cells has been reported previously (40). It is also possible that the secretory cytokine responses induced by both domains at an early time point were similar. However, the induction or inhibition of the development of Treg cells could be a late response shown by these two domains.

Along with the induction of Treg cells, Gal-9 has also been shown to suppress the generation of Th17 cells (41). We also observed significantly lower IL-17 levels after full-length Gal-9 treatment as compared with the unstimulated control. Full-length Gal-9 treatment also resulted in higher IL-4R gene expression in CD4 T cells. CD44, one of the ligands of Gal-9, has been also shown to influence IL-4R expression, thereby participating in TH1/TH2 differentiation (42). All three forms of Gal-9 were also shown to induce Ki-67 expression on CD4 and CD8 T cells, suggesting their role in T-cell proliferation. Gal-9 has been previously reported to expand CD4 and CD8 T cells by promoting cell division (22). Full-length galectin-9 was observed to result in a lesser effect on immune functionalities in our study. Full-length Gal-9 is likely to be present in higher concentrations during early infection before full-fledge inflammation sets in. IL-4R upregulation also suggests its primary role during early responses contributing to macrophage activation and might drive Th2-directed immune responses (43). It is likely that these differential functions are mediated by different receptors present on the PBMCs of the samples used for the assay. It is required to study different receptors and their downstream pathways involved in mediating these different functionalities of individual domains of Gal-9. It is also likely that the expression of these receptors might vary depending on the stage of HIV infection as well as ART duration and the extent of the immune reconstitution. We restricted our study to the patients on ART for 1 year to avoid variations. However, future *ex-vivo* as well as *in-vivo* studies are required on patients on ART for a longer duration of time to confirm the differences in functionalities mediated by them as well as to understand their role in the elimination of the HIV reservoir or mediating adverse events by releasing proinflammatory cytokines.

## Conclusion

We report, hereby, the induction of HIV reactivation by the N- and C-terminal domains of galectin-9 in addition to the full-length Gal-9 for the first time. The ability of the terminal domains to induce HIV reactivation was similar to that of the full-length protein indicating their possible use for the shock-and-kill strategy. The two domains elicited higher cytokine responses than the full-length protein possibly indicating their pronounced role in affecting T-cell functionality. N-CRD was found to induce the development of Treg cells and might be responsible for the anti-inflammatory properties of Gal-9 protein, whereas C-CRD inhibited the induction of Treg cells and might contribute to proinflammatory conditions induced by Gal-9. CD4 cell phenotypes responsible for IL-10 secretory response by the different forms were found to be different, indicating that differences in their functionalities could be due to differences in their target cells. Future studies are required to understand the role of the full-length versus individual CRDs in eliminating the HIV reservoir after its inducement.

## Data availability statement

The original contributions presented in the study are included in the article/supplementary material. Further inquiries can be directed to the corresponding author.

## Ethics statement

The studies involving human participants were reviewed and approved by the ICMR-National AIDS Research Institute Ethics Committee. The patients/participants provided their written informed consent to participate in this study.

## Author contributions

Conceptualization, funding acquisition, and writing—original draft: AS. Methodology and data analysis: MB, JS, and SD. All authors contributed to the article and approved the submitted version.

## References

[1] Wang WH Lin CY Chang MR Urbina AN Assavalapsakul W Thitithanyanont A . The role of galectins in virus infection - a systemic literature review. J Microbiol Immunol Infect (2020) 53(6):925–35. doi: 10.1016/j.jmii.2019.09.005 31630962

[2] Shete A Bichare S Pujari V Virkar R Thakar M Ghate M . Elevated levels of galectin-9 but not osteopontin in HIV and tuberculosis infections indicate their roles in detecting MTB infection in HIV infected individuals. Front Microbiol (2020) 11:1685. doi: 10.3389/fmicb.2020.01685 32765475 PMC7380070

[3] Tandon R Chew GM Byron MM Borrow P Niki T Hirashima M . Galectin-9 is rapidly released during acute HIV-1 infection and remains sustained at high levels despite viral suppression even in elite controllers. AIDS Res Hum Retroviruses (2014) 30(7):654–64. doi: 10.1089/aid.2014.0004 PMC4077009 24786365

[4] Abdel-Mohsen M Chavez L Tandon R Chew GM Deng X Danesh A . Human galectin-9 is a potent mediator of HIV transcription and reactivation. PloS Pathog (2016) 12(6):e1005677. doi: 10.1371/journal.ppat.1005677 27253379 PMC4890776

[5] Shete A Dhayarkar S Dhamanage A Kulkarni S Ghate M Sangle S . Possible role of plasma galectin-9 levels as a surrogate marker of viremia in HIV infected patients on antiretroviral therapy in resource-limited settings. AIDS Res Ther (2020) 17(1):43. doi: 10.1186/s12981-020-00298-9 32678033 PMC7364535

[6] Colomb F Giron LB Premeaux TA Mitchell BI Niki T Papasavvas E . Galectin-9 mediates HIV transcription by inducing TCR-dependent ERK signaling. Front Immunol (2019) 10:267. doi: 10.3389/fimmu.2019.00267 30842775 PMC6391929

[7] Bi S Hong PW Lee B Baum LG . Galectin-9 binding to cell surface protein disulfide isomerase regulates the redox environment to enhance T-cell migration and HIV entry. Proc Natl Acad Sci USA (2011) 108(26):10650–5. doi: 10.1073/pnas.1017954108 PMC3127870 21670307

[8] Elahi S Niki T Hirashima M Horton H . Galectin-9 binding to Tim-3 renders activated human CD4+ T cells less susceptible to HIV-1 infection. Blood (2012) 119(18):4192–204. doi: 10.1182/blood-2011-11-389585 PMC3359739 22438246

[9] Merani S Chen W Elahi S . The bitter side of sweet: the role of galectin-9 in immunopathogenesis of viral infections. Rev Med Virol (2015) 25(3):175–86. doi: 10.1002/rmv.1832 25760439

[10] Oomizu S Arikawa T Niki T Kadowaki T Ueno M Nishi N . Galectin-9 suppresses Th17 cell development in an IL-2-dependent but Tim-3-independent manner. Clin Immunol (2012) 143(1):51–8. doi: 10.1016/j.clim.2012.01.004 22341088

[11] Tureci O Schmitt H Fadle N Pfreundschuh M Sahin U . Molecular definition of a novel human galectin which is immunogenic in patients with hodgkin's disease. J Biol Chem (1997) 272(10):6416–22. doi: 10.1074/jbc.272.10.6416 9045665

[12] Matsushita N Nishi N Seki M Matsumoto R Kuwabara I Liu FT . Requirement of divalent galactoside-binding activity of ecalectin/galectin-9 for eosinophil chemoattraction. J Biol Chem (2000) 275(12):8355–60. doi: 10.1074/jbc.275.12.8355 10722666

[13] Li Y Feng J Geng S Geng S Wei H Chen G . The n- and c-terminal carbohydrate recognition domains of galectin-9 contribute differently to its multiple functions in innate immunity and adaptive immunity. Mol Immunol (2011) 48(4):670–7. doi: 10.1016/j.molimm.2010.11.011 21146220

[14] John S Mishra R . Galectin-9: From cell biology to complex disease dynamics. J Biosci (2016) 41(3):507–34. doi: 10.1007/s12038-016-9616-y 27581941

[15] Iwasaki-Hozumi H Chagan-Yasutan H Ashino Y Hattori T . Blood levels of galectin-9, an immuno-regulating molecule, reflect the severity for the acute and chronic infectious diseases. Biomolecules (2021) 11(3):430. doi: 10.3390/biom11030430 33804076 PMC7998537

[16] Ju SM Song HY Lee JA Lee SJ Choi SY Park J . Extracellular HIV-1 tat up-regulates expression of matrix metalloproteinase-9 *via* a MAPK-NF-kappaB dependent pathway in human astrocytes. Exp Mol Med (2009) 41(2):86–93. doi: 10.3858/emm.2009.41.2.011 19287189 PMC2679334

[17] Misse D Esteve PO Renneboog B Vidal M Cerutti M St Pierre Y . HIV-1 glycoprotein 120 induces the MMP-9 cytopathogenic factor production that is abolished by inhibition of the p38 mitogen-activated protein kinase signaling pathway. Blood (2001) 98(3):541–7. doi: 10.1182/blood.V98.3.541 11468147

[18] Suryawanshi P Godbole S Pawar J Thakar M Shete A . Higher expression of human telomerase reverse transcriptase in productively-infected CD4 cells possibly indicates a mechanism for persistence of the virus in HIV infection. Microbiol Immunol (2018) 62(5):317–26. doi: 10.1111/1348-0421.12585 29577368

[19] Wu C Thalhamer T Franca RF Xiao S Wang C Hotta C . Galectin-9-CD44 interaction enhances stability and function of adaptive regulatory T cells. Immunity (2014) 41(2):270–82. doi: 10.1016/j.immuni.2014.06.011 PMC4219323 25065622

[20] Zhu C Anderson AC Schubart A Xiong H Imitola J Khoury SJ . The Tim-3 ligand galectin-9 negatively regulates T helper type 1 immunity. Nat Immunol (2005) 6(12):1245–52. doi: 10.1038/ni1271 16286920

[21] Padilla ST Niki T Furushima D Bai G Chagan-Yasutan H Telan EF . Plasma levels of a cleaved form of galectin-9 are the most sensitive biomarkers of acquired immune deficiency syndrome and tuberculosis coinfection. Biomolecules (2020) 10(11):1495. doi: 10.3390/biom10111495 33143141 PMC7693693

[22] Gooden MJ Wiersma VR Samplonius DF Gerssen J van Ginkel RJ Nijman HW . Galectin-9 activates and expands human T-helper 1 cells. PloS One (2013) 8(5):e65616. doi: 10.1371/journal.pone.0065616 23741502 PMC3669208

[23] Lhuillier C Barjon C Niki T Gelin A Praz F Morales O . Impact of exogenous galectin-9 on human T cells: CONTRIBUTION OF THE T CELL RECEPTOR COMPLEX TO ANTIGEN-INDEPENDENT ACTIVATION BUT NOT TO APOPTOSIS INDUCTION. J Biol Chem (2015) 290(27):16797–811. doi: 10.1074/jbc.M115.661272 PMC4505427 25947381

[24] Yang R Sun L Li CF Wang YH Yao J Li H . Galectin-9 interacts with PD-1 and TIM-3 to regulate T cell death and is a target for cancer immunotherapy. Nat Commun (2021) 12(1):832. doi: 10.1038/s41467-021-21099-2 33547304 PMC7864927

[25] Finzi D Blankson J Siliciano JD Margolick JB Chadwick K Pierson T . Latent infection of CD4+ T cells provides a mechanism for lifelong persistence of HIV-1, even in patients on effective combination therapy. Nat Med (1999) 5(5):512–7. doi: 10.1038/8394 10229227

[26] Baxter AE O'Doherty U Kaufmann DE . Beyond the replication-competent HIV reservoir: transcription and translation-competent reservoirs. Retrovirology (2018) 15(1):18. doi: 10.1186/s12977-018-0392-7 29394935 PMC5797386

[27] Gleason MK Lenvik TR McCullar V Felices M O'Brien MS Cooley SA . Tim-3 is an inducible human natural killer cell receptor that enhances interferon gamma production in response to galectin-9. Blood (2012) 119(13):3064–72. doi: 10.1182/blood-2011-06-360321 PMC3321868 22323453

[28] Golden-Mason L McMahan RH Strong M Reisdorph R Mahaffey S Palmer BE . Galectin-9 functionally impairs natural killer cells in humans and mice. J Virol (2013) 87(9):4835–45. doi: 10.1128/JVI.01085-12 PMC3624298 23408620

[29] Meggyes M Miko E Polgar B Bogar B Farkas B Illes Z . Peripheral blood TIM-3 positive NK and CD8+ T cells throughout pregnancy: TIM-3/galectin-9 interaction and its possible role during pregnancy. PloS One (2014) 9(3):e92371. doi: 10.1371/journal.pone.0092371 24651720 PMC3961322

[30] Steelman AJ Li J . Astrocyte galectin-9 potentiates microglial TNF secretion. J Neuroinflammation. (2014) 11:144. doi: 10.1186/s12974-014-0144-0 25158758 PMC4158089

[31] Jankovic D Kugler DG Sher A . IL-10 production by CD4+ effector T cells: a mechanism for self-regulation. Mucosal Immunol (2010) 3(3):239–46. doi: 10.1038/mi.2010.8 PMC4105209 20200511

[32] Noble A Giorgini A Leggat JA . Cytokine-induced IL-10-secreting CD8 T cells represent a phenotypically distinct suppressor T-cell lineage. Blood (2006) 107(11):4475–83. doi: 10.1182/blood-2005-10-3994 16467201

[33] Suyama M Daikoku E Goto T Sano K Morikawa Y . Reactivation from latency displays HIV particle budding at plasma membrane, accompanying CD44 upregulation and recruitment. Retrovirology (2009) 6:63. doi: 10.1186/1742-4690-6-63 19594910 PMC2714482

[34] Shete A Suryawanshi P Godbole S Pawar J Paranjape R Thakar M . HIV-Infected CD4+ T cells use T-bet-dependent pathway for production of IL-10 upon antigen recognition. Scand J Immunol (2016) 83(4):288–96. doi: 10.1111/sji.12422 27028319

[35] Kaneko N Kurata M Yamamoto T Morikawa S Masumoto J . The role of interleukin-1 in general pathology. Inflammation Regen. (2019) 39:12. doi: 10.1186/s41232-019-0101-5 PMC6551897 31182982

[36] Ochoa-Reparaz J Rynda A Ascon MA Yang X Kochetkova I Riccardi C . IL-13 production by regulatory T cells protects against experimental autoimmune encephalomyelitis independently of autoantigen. J Immunol (2008) 181(2):954–68. doi: 10.4049/jimmunol.181.2.954 PMC2599928 18606647

[37] Eller K Wolf D Huber JM Metz M Mayer G McKenzie AN . IL-9 production by regulatory T cells recruits mast cells that are essential for regulatory T cell-induced immune suppression. J Immunol (2011) 186(1):83–91. doi: 10.4049/jimmunol.1001183 21115728 PMC3227733

[38] Bajnok A Ivanova M Rigo J Jr. Toldi G . The distribution of activation markers and selectins on peripheral T lymphocytes in preeclampsia. Mediators Inflamm (2017) 2017:8045161. doi: 10.1155/2017/8045161 28555090 PMC5438859

[39] O'Brien CA Batista SJ Still KM Harris TH . IL-10 and ICOS differentially regulate T cell responses in the brain during chronic toxoplasma gondii infection. J Immunol (2019) 202(6):1755–66. doi: 10.4049/jimmunol.1801229 PMC6401250 30718297

[40] Jung MK Lee JS Kwak JE Shin EC . Tumor necrosis factor and regulatory T cells. Yonsei Med J (2019) 60(2):126–31. doi: 10.3349/ymj.2019.60.2.126 PMC6342721 30666833

[41] Seki M Oomizu S Sakata KM Sakata A Arikawa T Watanabe K . Galectin-9 suppresses the generation of Th17, promotes the induction of regulatory T cells, and regulates experimental autoimmune arthritis. Clin Immunol (2008) 127(1):78–88. doi: 10.1016/j.clim.2008.01.006 18282810

[42] Yang C Lin J Liang H Xue L Kwart A Jiang M . CD44 v5 domain inhibition represses the polarization of Th2 cells by interfering with the IL-4/IL-4R signaling pathway. Immunol Cell Biol (2022) 100(1):21–32. doi: 10.1111/imcb.12491 34219288

[43] Gordon S Martinez FO . Alternative activation of macrophages: mechanism and functions. Immunity (2010) 32(5):593–604. doi: 10.1016/j.immuni.2010.05.007 20510870

